# Characterizing clinical risk profiles of major complications in type 2 diabetes mellitus using deep learning algorithms

**DOI:** 10.3389/fendo.2025.1657366

**Published:** 2025-09-10

**Authors:** Haochen Liu, Xiaomiao Li, Ke Shi, Fengyu Lei, Ziyan Wang, Ziyuan Gao, Yunxi Liu, Jing Zhu, Jiajia Zhai, Yi Zhang, Xinyu Li, Shiyu Wang, Yu Niu, Louyan Ma, Tianxiao Zhang

**Affiliations:** ^1^ Department of Epidemiology and Biostatistics, School of Public Health, Xi’an Jiaotong University Health Science Center, Xi’an, China; ^2^ Department of General Practice, Xi’an No.9 Hospital, Xi’an, Shaanxi, China; ^3^ Department of Endocrinology, Xijing Hospital, Air Force Medical University, Xi’an, Shaanxi, China; ^4^ Department of Biological Sciences, Mellon College of Science, Carnegie Mellon University, Pittsburgh, PA, United States; ^5^ Computational Biology Department, School of Computer Science, Carnegie Mellon University, Pittsburgh, PA, United States; ^6^ Department of Endocrinology, Xi’an No.9 Hospital, Xi’an, Shaanxi, China; ^7^ Department of Graduate School, Yanan Medical College of Yanan University, Yanan, Shaanxi, China

**Keywords:** Type 2 diabetes mellitus (T2DM), diabetic complications, SHAP (Shapley Additive explanation), machine learning, risk factors

## Abstract

**Objective:**

To develop a self-reportable risk assessment tool for elderly type 2 diabetes mellitus (T2DM) patients, evaluating risks of diabetic nephropathy (DN), retinopathy (DR), peripheral neuropathy (DPN), and diabetic foot (DF) using machine learning, thereby providing new insights and tools for the screening and intervention of these complications.

**Materials and methods:**

Data from 1,448 T2DM patients at Xi’an No.9 Hospital were used. After preprocessing, five machine learning algorithms (XGBoost, LightGBM, Random Forest, TabPFN, CatBoost) were applied. Models were trained on 70% of the data and evaluated on 30%, with performance assessed by multiple metrics and SHAP analysis for feature importance.

**Results:**

The analysis identified 33 risk factors, including 6 shared risk factors (UACR for DN and DR; diabetes duration for DR, DPN, and DF; IBILI for DF and DPN; history of DN for DR and DF; U-Cr for DR and DF; MCHC for DN and DPN) and 27 unique risk factors. Model performance was robust: for DN, TabPFN achieved an AUC of 0.905 and Random Forest an accuracy of 0.878; for DR, LightGBM attained an AUC of 0.794; for DPN, both TabPFN and CatBoost achieved a perfect recall of 1.000 and F1-score of 0.915; and for DF, LightGBM attaining the highest AUC of 0.704. SHAP analysis highlighted key features for each complication, such as UACR and Y-protein for DN, diabetes duration and TPOAB for DR, history of DN and IBILI for DF, and diabetes duration and SBP for DPN.

**Conclusion:**

This study employed interpretable machine learning to characterize risk factor profiles for multiple T2DM complications, identifying both common and distinct factors associated with major complications. The findings provide a foundation for exploring personalized risk management strategies and highlight the potential of data-driven approaches to inform early intervention research in T2DM complications.

## Introduction

Diabetes mellitus, especially type 2 diabetes (T2DM), represents a growing global health crisis, with 537 million individuals currently affected worldwide (1), and projections suggest this number will rise to 783 million by 2045 (2). Major complications such as diabetic nephropathy (DN), retinopathy (DR), peripheral neuropathy (DPN), and diabetic foot (DF) significantly associated with morbidity and mortality of patients with T2DM. DN affects approximately 30-40% of individuals with type 1 or type 2 diabetes, making it a leading cause of end-stage renal disease (ESRD) in developed countries (3). DR, a major cause of visual impairment and blindness, is similarly linked to diabetes (4). DPN prevalence increases with the duration of diabetes, and it is estimated that around 50% of patients with T2DM will develop neuropathy during their lifetime (5). DF is one of the most severe complications in diabetic patients, often leading to disability and death (6). Recent studies emphasize that elderly individuals with T2DM are particularly prone to significant declines in renal function, highlighting the importance of early interventions to manage complications effectively (7). Early detection is crucial, as timely interventions can prevent the progression of complications (8).

Accurately screening T2DM patients for the relevant complications remains a significant challenge. Conventional risk models based on logistic regression and Cox proportional hazards have been criticized for their limited ability to capture nonlinear relationships between risk factors (e.g., HbA1c, blood pressure, and lipid profiles) and complications (9). Recent studies have applied various machine learning techniques to evaluate the risk of DN. However, as highlighted by a review (10), most of these studies have only partially exploited the temporal factors in EHR data. Additionally, although the integration of omics data has shown potential to improve risk assessment, limitations such as small sample sizes and insufficient external validation still persist. Traditional single-complication models overlook shared pathophysiological pathways (e.g., hyperglycemia-induced endothelial dysfunction in DN, DR, and DPN) (11) and predominantly focus on individual complications, thereby limiting holistic management of T2DM—particularly problematic that almost 75% of patients have at least one additional comorbidity at the time of T2DM diagnosis and 44% have at least two comorbidities. Moreover, over 40% of those aged 60 and above have three or more long-term conditions (12). In a recent study, Ji et al. developed a machine learning model for type 1 diabetes (T1D) patients to self-identify risks of major complications (DR, DN, DPN), achieving strong internal and external validation performance (13). T2DM presents distinct challenges: higher prevalence, complex risk factors (e.g., obesity, insulin resistance) (1), and a greater need for scalable tools to evaluate the risk of multiple complications. This study integrates multiple machine learning algorithms to develop risk assessment models for various complications of T2DM, aiming to identify both shared and unique potential risk factors across different T2DM complications, thereby providing new insights and tools for the screening and intervention of these complications.

## Materials and methods

### Study participants

Clinical data for the 1,448 T2DM patients were retrospectively collected from Xi’an No.9 Hospital between January 2022 and December 2023, with input features (laboratory measures and clinical indicators) gathered during patients’ hospitalization and in the period after discharge. Participants were included in the study if they were aged 18 years or older, had a confirmed diagnosis of T2DM, and had complete clinical data. Participants were excluded if they had incomplete clinical data, missing information on any of the four aforementioned complications, or other primary causes of renal or vascular dysfunction. The detailed process of participant screening, including initial recruitment numbers and reasons for exclusion, is illustrated in **Supplementary Figure 1**. All participants were diagnosed with T2DM based on the American Diabetes Association (ADA) diagnostic criteria, with confirmation from two independent endocrinologists. The diagnosis was validated through clinical evaluation and laboratory findings, including HbA1c and fasting blood glucose levels, with a documented disease duration of at least one year. Diagnostic criteria for diabetic nephropathy (DN), retinopathy (DR), peripheral neuropathy (DPN), and diabetic foot (DF) were aligned with the China Guidelines for the Prevention and Treatment of Diabetes (2024 Edition) (14), with detailed criteria provided in **Supplementary Materials**. This study was approved by the Medical Biological Research Ethics Committee of Xi’an Ninth Hospital (Approval No.202516). All patient data were de-identified for confidentiality, with the study adhering to the Declaration of Helsinki; informed consent was waived for the retrospective, de-identified data as approved by the ethics committee.

### Features and data preprocessing

This study included a total of 129 features, derived from an initial set of 152 variables with strongly correlated ones (defined by a correlation coefficient threshold of |r| > 0.8) excluded through prior correlation analysis to avoid information redundancy, and these features were finally classified into three major groups: demographic indicators (age, sex, BMI, diabetes duration, etc.), biochemical markers (HbA1c, UACR, lipid profiles, etc.), and complication-related indices (TPOAB, D-Dimer, etc.). Categorical variables (e.g., smoking status, complication diagnoses) were encoded as binary or one-hot formats, while continuous variables (e.g., blood pressure, HbA1c, UACR) were standardized via Z-score transformation to normalize their scales for model training. All the features had missing rates <5%. Missing values were imputed using the Multiple Imputation by Chained Equations (MICE) method, a well-established technique. To assess the impact of MICE imputation on model performance, a complete-case analysis using a subset with no missing values is performed, applying the same algorithms as the main study and comparing AUC between complete-case and MICE-imputed datasets.

### Model construction and validation

The detailed process of model construction is shown in **Supplementary Figure 2**. To develop risk models for diabetic complications (DR, DN, DPN, and DF), five machine learning algorithms were selected based on their distinct strengths in handling clinical tabular data. For DF specifically, resampling and penalization were applied to address class imbalance. These algorithms include XGBoost (15) and LightGBM (16) for their efficiency in capturing non-linear relationships and interactions among risk factors. Random Forest (17) for its robustness in high-dimensional data and resistance to overfitting, CatBoost (18) for its superior handling of categorical variables without manual encoding, and TabPFN (19) a tabular foundation model, for its ability to generate synthetic data to augment small samples. In model construction, algorithm parameters followed conventional settings. For DF class imbalance, SMOTE oversampling for the minority class and class weight adjustment were applied. The dataset was randomly split into a training set (70%) and a test set (30%), with 5-fold cross-validation on the training set to ensure stability. Model performance was assessed using accuracy, precision, recall, F1-score, and ROC-AUC. SHapley Additive exPlanations (SHAP) analysis was specifically used with XGBoost to interpret the relative importance of key clinical features in assessing the risk factors of complications (20–22). Logistic regression was implemented as a reference to benchmark the five machine learning algorithms. It underwent the same preprocessing of variables and was evaluated using the same metrics to ensure direct comparability. All statistical analyses were performed using R 4.3.3 and Python 3.7.7. Details of the XGBoost algorithm and SHAP analysis are available at https://github.com/dmlc/xgboost and http://github.com/slundberg/shap, respectively.

## Results

### Clinical and demographic characteristics of patients

Data from 1,448 participants with 129 variables were collected for analysis (**Table 1**, **Supplementary Table S1**). Distributions of multiple variables were present and compared between the training (N = 1,013) and test set (N = 435). The median age was 54.0 years, with no significant difference between the two groups (*P* = 0.514). A majority of patients were male (66.9%), and distributions of smoking status, alcohol consumption, diabetes duration (median 8.0 years), waist circumference, BMI, and WHR were similar between the two datasets. Twenty variables in the dataset had missing rates less than 5%, with detailed information on variable missing rates (**Supplementary Table S2**).The prevalences of DN, DR, DF, and DPN in the T2DM patients were 23.3%, 27.2%, 2.8%, and 85.2%, respectively.

**Table 1:** Baseline clinical and demographic characteristics of patients in the overall, training and test set.

Characteristics	Levels	Overall	Training set	Test set	*P*-Value
Total		1448	1013	435	
Age, median (IQR)		54.0 (15.0)	54.0 (15.0)	53.0 (16.0)	0.514
Sex, n (%)	Female	479 (33.1)	342 (33.8)	137 (31.5)	0.436
	Male	969 (66.9)	671 (66.2)	298 (68.5)	
Educational level, n (%)	0	1 (0.1)		1 (0.2)	0.613
	1	52 (3.6)	38 (3.8)	14 (3.2)	
	2	163 (11.3)	111 (11.0)	52 (12.0)	
	3	324 (22.4)	227 (22.4)	97 (22.3)	
	4	371 (25.6)	266 (26.3)	105 (24.1)	
	5	537 (37.1)	371 (36.6)	166 (38.2)	
Smoking, n (%)	No	789 (54.5)	556 (54.9)	233 (53.6)	0.685
	Yes	659 (45.5)	457 (45.1)	202 (46.4)	
Alcohol, n (%)	No	1053 (72.7)	748 (73.8)	305 (70.1)	0.163
	Yes	395 (27.3)	265 (26.2)	130 (29.9)	
Family history, n (%)	No	725 (50.1)	512 (50.5)	213 (49.0)	0.622
	Yes	723 (49.9)	501 (49.5)	222 (51.0)	
Diabetes duration, median (IQR)		8.0 (10.0)	8.0 (10.0)	8.0 (10.0)	0.517
Waist, median (IQR)		90.0 (11.0)	90.0 (11.0)	90.0 (12.0)	0.891
BMI, median (IQR)		25.5 (4.3)	25.6 (4.1)	25.1 (4.4)	0.371
WHR, median (IQR)		0.9 (0.1)	0.9 (0.1)	0.9 (0.1)	0.485
SBP, median (IQR)		130.0 (20.0)	130.0 (20.0)	130.0 (20.0)	0.678
DBP, median (IQR)		80.0 (16.0)	80.0 (15.0)	80.0 (20.0)	0.411
DN, n (%)	No	1110 (76.7)	773 (76.3)	337 (77.5)	0.680
	Yes	338 (23.3)	240 (23.7)	98 (22.5)	
DR, n (%)	No	1054 (72.8)	728 (71.9)	326 (74.9)	0.254
	Yes	394 (27.2)	285 (28.1)	109 (25.1)	
DF, n (%)	No	1407 (97.2)	989 (97.6)	418 (96.1)	0.148
	Yes	41 (2.8)	24 (2.4)	17 (3.9)	
DPN, n (%)	No	214 (14.8)	146 (14.4)	68 (15.6)	0.604
	Yes	1234 (85.2)	867 (85.6)	367 (84.4)	

Bonferroni correction was applied for multiple comparisons, with a corrected significance threshold of *P* < 0.0031.

### Performance of machine learning models for T2DM complications

**Figure 1:**
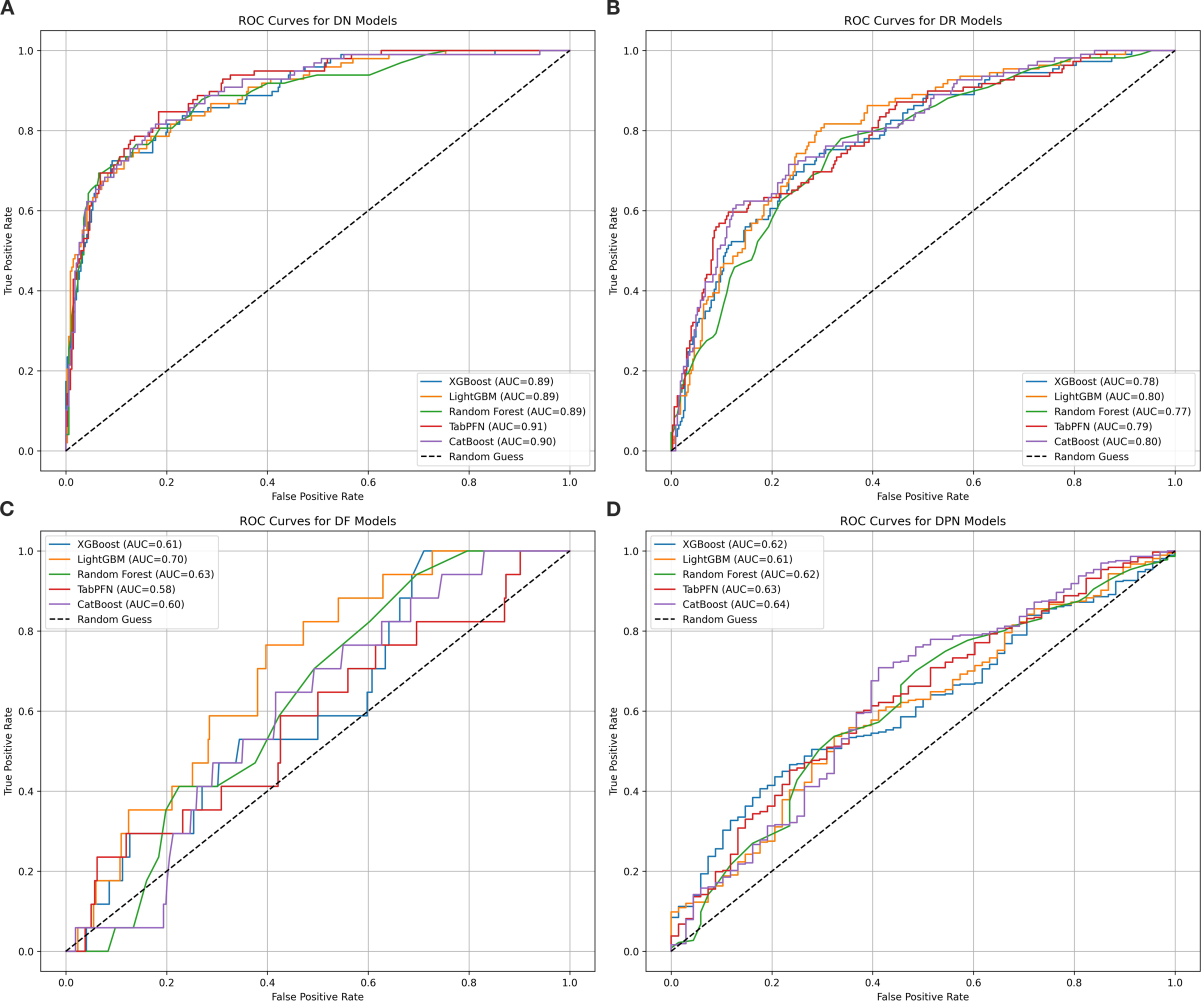
ROC curves for five machine learning models assessing four diabetic major complications. This figure presents receiver operating characteristic (ROC) curves evaluating the discriminative performance of five machine learning models (XGBoost, LightGBM, Random Forest, TabPFN, and CatBoost) in classifying patients with four diabetic major complications: diabetic nephropathy (DN, subplot **A**), retinopathy (DR, subplot **B**), foot (DF, subplot **C**), and peripheral neuropathy (DPN, subplot **D**). The algorithms are color-coded as blue, orange, green, red, and purple, respectively. AUC values quantify model performance, with higher values indicating better ability to distinguish complication types. For DN models, TabPFN achieves the highest AUC of 0.91, followed closely by CatBoost at 0.90, while the other three algorithms have an AUC of 0.89. In DR models, LightGBM and CatBoost both reach an AUC of 0.80. For DF models, LightGBM shows the best performance with an AUC of 0.70. In DPN models, CatBoost leads with an AUC of 0.64, and all algorithms surpass random performance (AUC > 0.5).

Machine learning models for four major complications (DN, DR, DF, and DPN) of T2DM were evaluated using five algorithms, with results in **Table 2** and **Figure 1**. The model performance for DN was the best among the four complications. Specifically, TabPFN achieved the highest area under the curve (AUC) of 0.905, Random Forest yielded the highest accuracy at 0.878, and XGBoost obtained the highest F1 score of 0.703. Among the models for DR, LightGBM achieved the highest AUC of 0.801, while TabPFN showed the highest accuracy of 0.805. For DF, after resampling and penalization, Random Forest and TabPFN achieved high accuracy (0.961), with LightGBM attaining the highest AUC, at 0.704. In the models of DPN, both TabPFN and CatBoost achieved perfect recall (1.000), along with the highest F1 scores (0.915) and Accuracy (0.844). Overall, TabPFN demonstrated robust performance across multiple diabetic complications, while other algorithms exhibited specific strengths in evaluating individual complications. Logistic regression was additionally evaluated for the four complications, with detailed metrics provided in **Supplementary Table S3**. The five machine learning algorithms (XGBoost, LightGBM, Random Forest, TabPFN, and CatBoost) consistently outperformed logistic regression across key metrics. AUC consistency between the 812-sample complete-case and MICE-imputed datasets across algorithms is shown in **Supplementary Table S4**.

**Table 2:** Metrics of model performance evaluated for risk assessment models for DN, DR, DF, and DPN based on five machine learning algorithms.

Complications	Model	Accuracy	Precision	Recall	F1-Score	AUC
DN	XGBoost	0.874	0.747	0.663	0.703	0.889
	LightGBM	0.871	0.756	0.633	0.689	0.892
	Random Forest	0.878	0.826	0.582	0.683	0.888
	TabPFN	0.871	0.750	0.643	0.692	0.905
	CatBoost	0.871	0.763	0.622	0.685	0.898
DR	XGBoost	0.779	0.594	0.376	0.461	0.782
	LightGBM	0.789	0.627	0.385	0.477	0.801
	Random Forest	0.772	0.619	0.239	0.344	0.768
	TabPFN	0.805	0.658	0.459	0.541	0.794
	CatBoost	0.795	0.685	0.339	0.454	0.800
DF	XGBoost	0.851	0.071	0.235	0.110	0.611
	LightGBM	0.945	–	–	–	0.704
	Random Forest	0.961	–	–	–	0.627
	TabPFN	0.961	–	–	–	0.579
	CatBoost	0.841	0.019	0.059	0.028	0.604
DPN	XGBoost	0.825	0.844	0.973	0.904	0.616
	LightGBM	0.834	0.845	0.984	0.909	0.606
	Random Forest	0.837	0.843	0.992	0.911	0.618
	TabPFN	0.844	0.844	1.000	0.915	0.632
	CatBoost	0.844	0.844	1.000	0.915	0.636

### Feature importance of the models for T2DM complications

**Figure 2:**
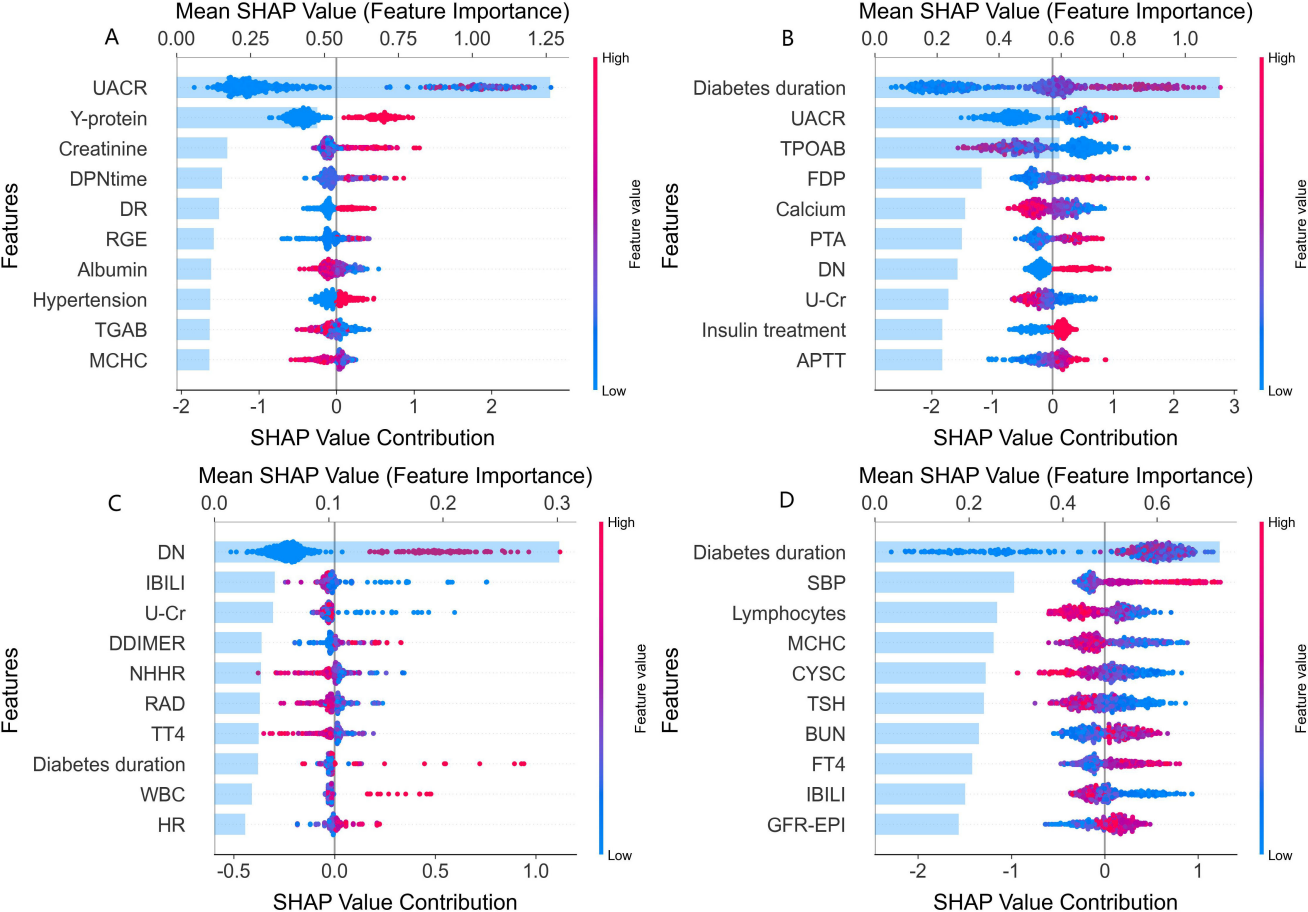
SHAP value analysis of clinical feature contributions to major complications. SHAP (SHapley Additive exPlanations) analysis plots depicting the contributions of various features to the different diabetic major complications. Each subplot **(A-D)** corresponds to a specific complication. In each plot, the x-axis represents the Shapley value contribution, reflecting the impact of each feature on the assessment. The y-axis lists the features, and the color of the points (blue to red) indicates the feature value (low to high). **(A)** Features influencing the assessment of diabetic nephropathy (DN), such as UACR, Y-protein, and creatinine. **(B)** Features for diabetic retinopathy (DR), including diabetes duration, UACR, and TPOAB. **(C)** Features related to diabetic foot (DF), like DN history (DN), IBILI, and U - Cr. **(D)** Features affecting diabetic peripheral neuropathy (DPN), such as diabetes duration, SBP, and lymphocytes. This visualization shows how much each feature impacts the complication assessment, with higher Shapley values indicating stronger impacts.

The top 10 most important features for the four major complications identified through SHAP analysis combined with the XGBoost model were summarized in **Figure 2**. For DN, UACR (Urinary Albumin-to-Creatinine Ratio) and Y-protein emerged as the most influential features, creatinine and DPNtime were also significant contributors. For DR, diabetes duration was the most critical factor, followed by UACR and TPOAB. Longer diabetes duration significantly increases the risk of DR, highlighting its central role in the risk model for retinopathy. For DF, the history of DN, indirect bilirubin (IBILI), and urinary creatinine (U-Cr) were key features in assessing the risk of diabetic foot complications. In the model of DPN, diabetes duration was the most important feature, followed by systolic blood pressure (SBP) and peripheral lymphocyte levels. The ranking of all variables for complications is provided in **Supplementary Table S5**.

### Shared and unique features for diabetic complications

**Figure 3:**
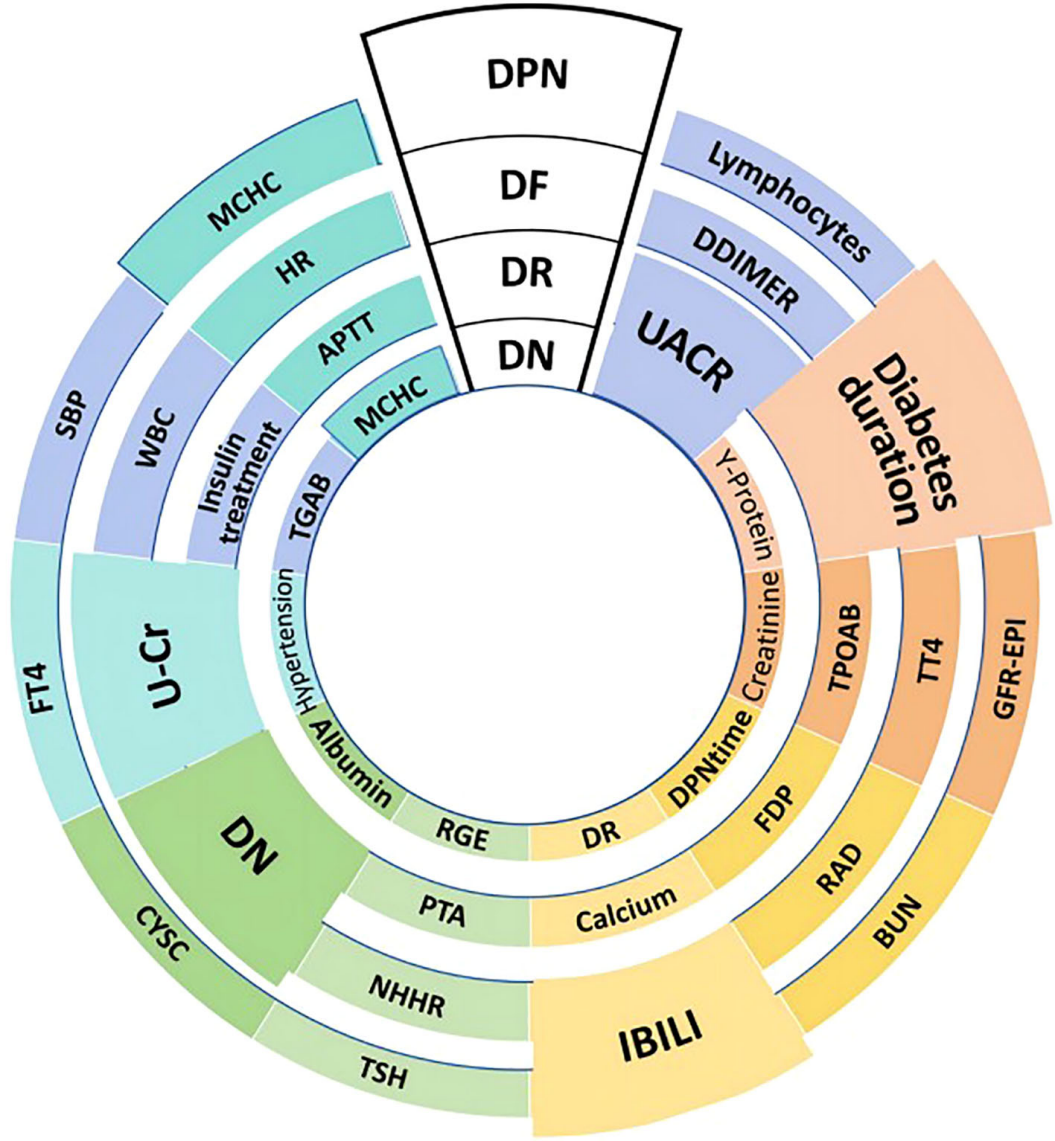
Shared and unique risk factors for T2DM-related major complications. This figure outlines the key influencing factors for four major complications in T2DM patients: diabetic nephropathy (DN), diabetic retinopathy (DR), diabetic foot (DF), and diabetic peripheral neuropathy (DPN). The size of each block reflects the relative importance of the corresponding risk factor. DR and DN are shown in two ways: DR at the top-middle is the disease itself; inner DR indicates the history of DR. Similarly, DN at the top-middle represents the disease, while inner DN serves as a risk factor which is the history of DN. Shared risk factors include UACR (Urine Albumin-to-Creatinine Ratio) for DN and DR, and diabetes duration for DR, DPN, and DF. Unique risk factors are Y-protein and creatinine for DN, TPOAB (Thyroid Peroxidase Antibody) for DR, D-Dimer for DF, and lymphocytes(peripheral lymphocyte levels) for DPN. Other factors indicated include BUN (Blood Urea Nitrogen), RAD (Right Atrial Diameter), and FDP (Fibrin Degradation Products). The figure also includes various laboratory parameters such as GFR-EPI (estimated glomerular filtration rate calculated using the EPI equation), TT4 (Total Thyroxine), TSH (Thyroid-Stimulating Hormone), APTT (Activated Partial Thromboplastin Time), HR (Heart Rate), MCHC (Mean Corpuscular Hemoglobin Concentration), and TGAB (Thyroglobulin Antibody).

The rank of the top 10 most important features for the four major complications was visualized in **Figure 3**. Six shared risk factors were identified, UACR emerged as a shared risk factor across DN and DR. Diabetes duration was a significant factor in DR, DPN, and DF, and notably was a top-ranking risk factor for DR. IBILI was identified as a shared risk factor in DF and DPN. The history of DN was a common risk factor in DR and DF. U-Cr was identified as a shared risk factor in DR and DF. MCHC was also found to be a shared factor for DN and DPN. In terms of unique risk factors, DN was associated with kidney-specific indices like Y-protein and creatinine; DR was linked to thyroid peroxidase antibody (TPOAB) and Fibrin Degradation Products(FPD); DF was affected by parameters like D-Dimer and Total Thyroxine(TT4). Notably, D-Dimer ranked prominently in the risk assessment of DF; DPN was related to peripheral lymphocyte levels and systolic blood pressure (SBP), among others.

## Discussion

In the present study, features that might assess the risk of major complications of T2DM were evaluated using a cross-sectional sample comprised of T2DM patients. Machine learning models were constructed and evaluated based on five algorithms, including XGBoost, LightGBM, Random Forest, TabPFN, and CatBoost, to depict the patterns and profiles of risk features for multiple T2DM complications.

SHAP analysis revealed distinct risk profiles for each complication, shedding light on unique underlying biological pathways. For DN, kidney-specific markers such as Y-protein and creatinine are strong risk factors, reflecting glomerular hyperfiltration and tubulointerstitial damage as key pathogenic mechanisms (23); these factors are associated with progressive renal fibrosis and loss of filtration integrity, critical hallmarks of DN (24, 25). DR risk rises with longer diabetes duration, making it central to retinopathy risk models. It is linked to TPOAB, indicating autoimmune processes may be associated with retinal vascular inflammation. Thyroid-mediated immune issues might be associated with endothelial injury and neovascularization in the retina. This suggests thyroid-related biomarkers could be important for assessing diabetic eye disease risk and need further study (26, 27). DF risk is linked to indirect bilirubin and urinary creatinine, with implications for metabolic and vascular factors related to the lower limbs indicated. This aligns with the pathophysiology of diabetic foot complications, where a prior history of DN suggests shared vascular pathogenesis, with an association with increased susceptibility to lower-limb issues. Abnormal levels of IBILI and changes in U-Cr may reflect underlying metabolic disturbances and renal/systemic vascular alterations, respectively (28, 29), both of which disrupt lower limb physiological processes, impair tissue integrity, and increase the risk of ulceration and infection in diabetic patients. DPN risk is associated with diabetes duration, systolic blood pressure (SBP), and peripheral lymphocyte levels, reflecting the combined association of prolonged hyperglycemia, hypertension, and lymphocyte-mediated inflammation with nerve injury (30). Diabetes duration is linked to cumulative hyperglycemic damage to peripheral nerves, while hypertension is associated with endoneurial ischemia and elevated lymphocytes promote axonal degeneration (31, 32). These factors are synergistically associated with exacerbate nerve fiber damage in DPN. Dynamic trade-offs between sensitivity and specificity across 99 cutoff values for each algorithm were further analyzed, with optimal thresholds identified based on maximum Youden’s Index to balance true detection of DPN cases and minimization of false positives in clinical practice (**Supplementary Figure 3**, **Supplementary Table S6**). Notably, MCHC was identified as a shared factor for DN and DPN. SHAP plots revealed that lower MCHC values are linked to higher SHAP values for both conditions, indicating that low MCHC may significantly increase complication risk. Additionally, a history of DN and urinary creatinine (U-Cr) emerged as shared risk factors across DR and DF, highlighting potential multi-comorbidity and overlapping metabolic mechanisms. Notably, markers like Y-protein and IBILI emerged as key risk factors, highlighting underrecognized associations that warrant further exploration of their clinical relevance and mechanisms. These findings not only reveal intrinsic connections between complications through shared risk factors, but also highlight each complication’s distinct pathophysiological mechanisms. These SHAP-identified features could translate to actionable steps: for DN and DR, integrate routine UACR monitoring into screenings to stratify high-risk patients for targeted renal/retinal assessments; for DR and DPN, long diabetes duration could trigger intensified monitoring, such as TPOAB testing for DR and neurological exams for DPN in those with longer duration; for DF, a history of DN could prompt quarterly foot inspections, with abnormal IBILI levels guiding peripheral circulation evaluations. Such steps link model insights to clinical workflows.

In this study, machine learning models were trained using five algorithms, each demonstrating distinct strengths. TabPFN and CatBoost showed unique advantages in handling categorical data. TabPFN achieved an AUC of 0.905 for DN. For DPN, while both TabPFN and CatBoost reached a perfect recall of 1.000 and an F1 score of 0.915, TabPFN still demonstrated notable performance. TabPFN’s innovative approach — generating synthetic tabular datasets via a pre-trained transformer-based neural network— addresses challenges in small or imbalanced datasets, By learning patterns from diverse tabular data during pre-training, it extrapolates effectively to limited samples, while its attention mechanism captures complex feature interactions, enhancing performance across complications like DN (AUC = 0.905) and DPN (recall=1.000) (19). Random Forest proved effective for class-imbalanced datasets (17), achieving the highest accuracy of 0.878 for DN. XGBoost stood out for its interpretability (15), attaining an F1 score of 0.703 for DN and providing clear explanations via feature importance scores. Notably, LightGBM also demonstrated the highest AUC of 0.704 for DF, outperforming other models. These findings highlight the efficiency of various algorithms in processing different data types and the importance of selecting the appropriate algorithm based on the dataset and problem characteristics. The strengths of these algorithms collectively enhance the evaluative accuracy and reliability of models in assessing the risk of diabetic major complications. By leveraging these algorithms, researchers and clinicians can develop more effective risk assessment tools to improve patient outcomes and manage the complex landscape of diabetes complications more efficiently.

Contrasting with prior research focused on single-complication risk assessment (10, 33), a holistic risk assessment framework is addressed in this study for T2DM. Unlike models developed for T1D (13, 34), T2D-specific variables (e.g., BMI, APOA1) are incorporated in the present study. With the higher prevalence of multiple long-term complications in elderly T2DM patients—where integrated risk management is essential—reflected. Notably, comparisons with logistic regression further confirm the added value of the machine learning approach. Logistic regression was observed to perform poorly across all four complications, particularly in capturing complex relationships between risk factors. In contrast, the five machine learning algorithms were observed to consistently achieve higher accuracy, AUC, and F1-score, demonstrating superior discriminative ability and robustness—this is particularly important for complications with complex associations, where traditional linear models struggle to perform. Thus, the machine learning models are provided as a more reliable tool for clinical risk assessment. By highlighting shared risk factors such as UACR and diabetes duration, calls for comprehensive strategies to address the interconnectedness of diabetic complication risks are aligned with, particularly relevant given that over 40% of T2DM patients aged ≥60 years have three or more comorbidities, necessitating risk stratification that transcends isolated organ-specific screening (35, 36).

This study has suffered from several limitations. The single-center (37), cross-sectional design may limit its generalizability. Despite the aforementioned limitations, key indicators of our study sample—including gender distribution, blood pressure levels, BMI, as well as the prevalence of DN and DR—showed no significant differences from the national average, which supports the rationality of the data to a certain extent. Due to low DF event numbers, most models lack complete Precision, Recall, and F1-Score, though resampling and algorithm adjustments were applied, with XGBoost and CatBoost yielding full metrics to support DF evaluation. For DPN, despite its high prevalence (85.2%), robust validation through 5-fold cross-validation, multi-metric evaluation, and SHAP-identified risk factors consistent with clinical pathophysiology support the reliability of model performance.

In addition, the lack of genetic, lifestyle, and omics data, as well as temporal variables and behavioral data such as medication adherence, may restrict the discovery of more risk markers and limit the models’ capacity to capture real-world dynamic risk patterns. Future research could focus on: increasing sample size and multi-center validation to enhance generalizability; integrating longitudinal data to capture temporal changes in risk factors; exploring shared risk pathways among complications; And applying stacked model approaches to integrate strengths of individual algorithms, potentially improving assessment performance for multi-comorbidity scenarios (38).

In conclusion, this study depicted the profiles of risk factors for multiple T2DM complications using interpretable machine learning algorithms. Several shared and unique risk factors for T2DM major complications were identified and reported. These insights lay the groundwork for future studies to validate risk stratification tools in multi-center cohorts, with the ultimate goal of supporting personalized risk management and data-driven early interventions.

## Data Availability

The raw data supporting the conclusions of this article will be made available by the authors, without undue reservation.

## Supplementary material

Supplementary Figure 1 Flowchart of Participant Inclusion and Exclusion. This flowchart illustrates the process of participant selection for the study. Initially, 2000 participants were screened. After applying the inclusion and exclusion criteria, 552 participants were excluded. These exclusions were categorized into three main reasons: 300 participants had missing data for more than 5% of the variables, 200 were either non-T2DM patients or under 18 years of age, and 52 were excluded due to other specified criteria. Eventually, 1448 participants met all the requirements and were included in the final analysis.

Supplementary Figure 2 Machine Learning Pipeline for T2DM Major Complication Risk Assessment. This figure presents the research workflow for analyzing major complications in T2DM patients. It begins with data collection, where inclusion and exclusion criteria are set to select data from 1,448 T2DM patients at Xi’an No.9 Hospital. Then the data preprocessing involves handling missing data using the MICE method and splitting the dataset into training and testing sets. In the model construction stage, five algorithms—XGBoost, LightGBM, Random Forest, CatBoost, and TabPFN—are applied and trained on the training set, with the test set reserved for evaluation. Finally, the model is evaluated using multiple metrics, including AUROC, Accuracy, Sensitivity, Specificity, and F1-score, along with SHAP analysis for feature importance.

Supplementary Figure 3 DPN Models Cutoff Analysis. This figure illustrates the dynamic trade-offs between sensitivity and specificity across 99 cutoff values for each algorithm, with optimal thresholds identified based on the maximum Youden’s Index to balance the true detection of DPN cases and the minimization of false positives in clinical practice. As shown in the three sub-plots, the top chart visualizes how the sensitivity of XGBoost, LightGBM, Random Forest, TabPFN, and CatBoost models changes with cutoff values when assessing DPN, reflecting their capacity to correctly identify true DPN-positive cases. The middle plot presents the variation of specificity across cutoff values, indicating the models’ ability to rule out false positives. The bottom sub-plot depicts Youden’s Index fluctuations, where peaks (e.g., the marked optimal values for each model in the legend) correspond to the cutoff points that best balance sensitivity and specificity, guiding the selection of thresholds for effective DPN diagnosis in real-world clinical scenarios.
