# Supplementary figures and images for: Characterizing clinical risk profiles of major complications in type 2 diabetes mellitus using deep learning algorithms
Source: Front Endocrinol (Lausanne). 2025 Sep 10;16:1657366. doi: 10.3389/fendo.2025.1657366 (PMC12457174; doi:10.3389/fendo.2025.1657366)

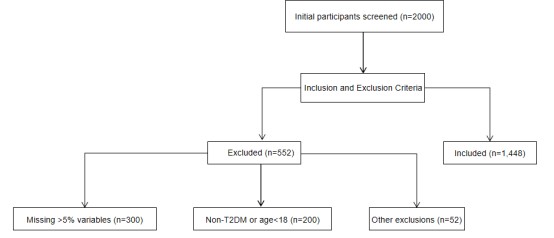

Supplement: Supplementary Figure 1 — Flowchart of Participant Inclusion and Exclusion. This flowchart illustrates the process of participant selection for the study. Initially, 2000 participants were screened. After applying the inclusion and exclusion criteria, 552 participants were excluded. These exclusions were categorized into three main reasons: 300 participants had missing data for more than 5% of the variables, 200 were either non-T2DM patients or under 18 years of age, and 52 were excluded due to other specified criteria. Eventually, 1448 participants met all the requirements and were included in the final analysis. [file Image1.jpeg]

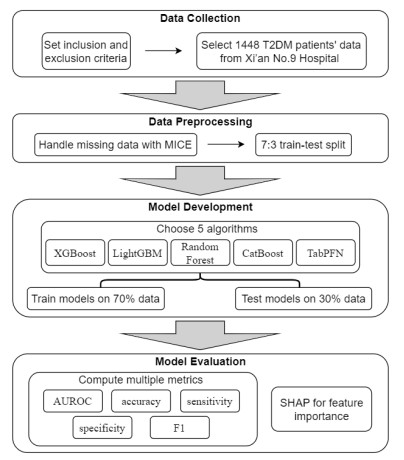

Supplement: Supplementary Figure 2 — Machine Learning Pipeline for T2DM Major Complication Risk Assessment. This figure presents the research workflow for analyzing major complications in T2DM patients. It begins with data collection, where inclusion and exclusion criteria are set to select data from 1,448 T2DM patients at Xi’an No.9 Hospital. Then the data preprocessing involves handling missing data using the MICE method and splitting the dataset into training and testing sets. In the model construction stage, five algorithms—XGBoost, LightGBM, Random Forest, CatBoost, and TabPFN—are applied and trained on the training set, with the test set reserved for evaluation. Finally, the model is evaluated using multiple metrics, including AUROC, Accuracy, Sensitivity, Specificity, and F1-score, along with SHAP analysis for feature importance. [file Image2.jpeg]

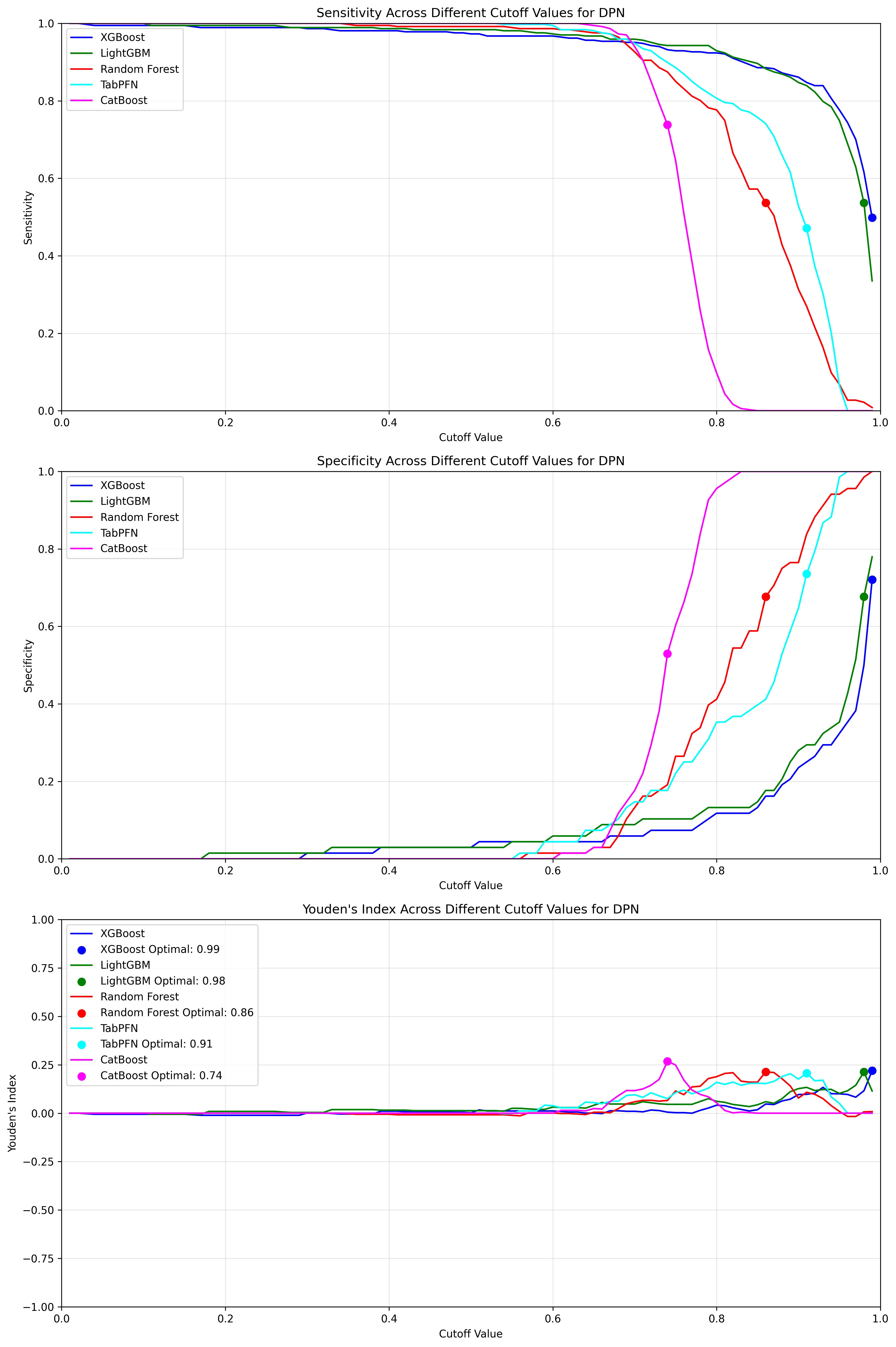

Supplement: Supplementary Figure 3 — DPN Models Cutoff Analysis. This figure illustrates the dynamic trade-offs between sensitivity and specificity across 99 cutoff values for each algorithm, with optimal thresholds identified based on the maximum Youden’s Index to balance the true detection of DPN cases and the minimization of false positives in clinical practice. As shown in the three sub-plots, the top chart visualizes how the sensitivity of XGBoost, LightGBM, Random Forest, TabPFN, and CatBoost models changes with cutoff values when assessing DPN, reflecting their capacity to correctly identify true DPN-positive cases. The middle plot presents the variation of specificity across cutoff values, indicating the models’ ability to rule out false positives. The bottom sub-plot depicts Youden’s Index fluctuations, where peaks (e.g., the marked optimal values for each model in the legend) correspond to the cutoff points that best balance sensitivity and specificity, guiding the selection of thresholds for effective DPN diagnosis in real-world clinical scenarios. [file Image3.jpeg]
